# Bis[μ-bis­(diphenyl­phosphan­yl)methane-κ^2^ *P*:*P*′](μ-1-ethyl­thio­urea-κ^2^ *S*:*S*)bis­[iodidocopper(I)] acetonitrile sesquisolvate

**DOI:** 10.1107/S1600536812050970

**Published:** 2012-12-22

**Authors:** Ruthairat Nimthong, Yupa Wattanakanjana, Chaveng Pakawatchai

**Affiliations:** a Department of Chemistry and Center of Excellence for Innovation in Chemistry, Faculty of Science, Prince of Songkla University, Hat Yai, Songkhla 90112, Thailand; b Department of Chemistry, Faculty of Science, Prince of Songkla University, Hat Yai, Songkhla 90112, Thailand

## Abstract

In the dinuclear title compound, [Cu_2_I_2_(C_3_H_8_N_2_S)(C_25_H_22_P_2_)_2_]·1.5CH_3_CN, each Cu^I^ atom exhibits a distorted tetra­hedral coordination with two P atoms from two bis­(diphenyl­phosphan­yl)methane (dppm) ligands, one metal-bridging S atom from the 1-ethyl­thio­urea (ettu) ligand and one iodide ion. The dppm ligand and the bridging S atom of the ettu ligand force the two copper atoms into close proximity, leading to the formation of a close intra­molecular Cu⋯Cu contact [3.3747 (17) Å]. The conformation of the dimeric complex is such that the two dppm ligands are located on one side of the dinuclear metal complex, while the two iodine atoms are pointed towards the other side of the complex, a conformation that is stabilized by two intra­molecular N—H⋯I hydrogen bonds between the ettu NH_2_ and NHEt moieties and the I atoms. Another pair of symmetry-equivalent N—H⋯I hydrogen bonds is established between neighboring mol­ecules across an inversion center, linking mol­ecules into dimers. The dimers are connected with each other and with the inter­stitial acetonitrile solvent mol­ecules *via* a range of weaker C—H⋯I and C—H⋯S inter­actions and through weak C—H⋯π inter­actions, leading to the formation of a three-dimensional network. One of the acetonitrile solvent mol­ecules is disordered in a 1:1 ratio across a crystallographic inversion center.

## Related literature

For potential applications of related complexes, see: Isab *et al.* (2010 ▶); Safin *et al.* (2010 ▶). For examples of dppm as a chelating ligand, see: Yang *et al.* (2000 ▶); Liaw *et al.* (2005 ▶); Jin *et al.* (2009 ▶). For relevant examples of discrete complexes, see: Colacio *et al.* (1997 ▶); Yam *et al.* (2001 ▶); Zhou *et al.* (2001 ▶); Nimthong *et al.* (2008 ▶); Pakawatchai *et al.* (2012 ▶).
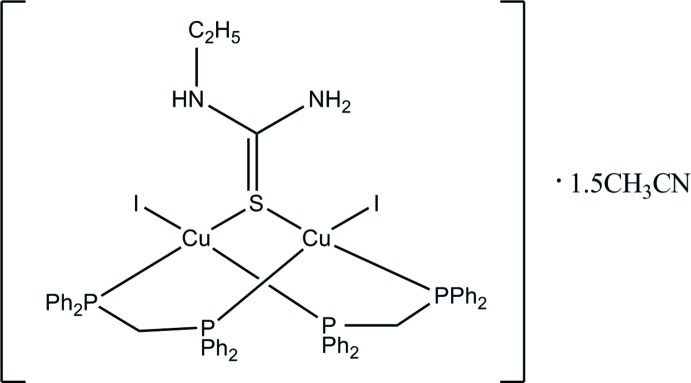


## Experimental

### 

#### Crystal data

[Cu_2_I_2_(C_3_H_8_N_2_S)(C_25_H_22_P_2_)_2_]·1.5C_2_H_3_N
*M* *_r_* = 1315.37
Monoclinic, 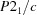

*a* = 13.7751 (6) Å
*b* = 24.5147 (11) Å
*c* = 18.0172 (8) Å
β = 111.720 (1)°
*V* = 5652.3 (4) Å^3^
*Z* = 4
Mo *K*α radiation
μ = 2.03 mm^−1^
*T* = 100 K
0.28 × 0.17 × 0.10 mm

#### Data collection

Bruker SMART CCD diffractometer
Absorption correction: multi-scan (*SADABS*; Bruker, 2003 ▶) *T*_min_ = 0.670, *T*_max_ = 0.821
53066 measured reflections
10863 independent reflections
9108 reflections with *I* > 2σ(*I*)
*R*_int_ = 0.054

#### Refinement

*R*[*F* ^2^ > 2σ(*F* ^2^)] = 0.046
*wR*(*F* ^2^) = 0.101
*S* = 1.05
10863 reflections
643 parameters
3 restraints
H atoms treated by a mixture of independent and constrained refinement
Δρ_max_ = 0.84 e Å^−3^
Δρ_min_ = −0.55 e Å^−3^

### 

Data collection: *SMART* (Bruker, 1998 ▶); cell refinement: *SAINT* (Bruker, 2003 ▶); data reduction: *SAINT*; program(s) used to solve structure: *SHELXS97* (Sheldrick, 2008 ▶); program(s) used to refine structure: *SHELXL97* (Sheldrick, 2008 ▶); molecular graphics: *Mercury* (Macrae *et al.*, 2008 ▶); software used to prepare material for publication: *SHELXL97* and *publCIF* (Westrip, 2010 ▶).

## Supplementary Material

Crystal structure: contains datablock(s) I, global. DOI: 10.1107/S1600536812050970/zl2525sup1.cif

Structure factors: contains datablock(s) I. DOI: 10.1107/S1600536812050970/zl2525Isup2.hkl

Additional supplementary materials:  crystallographic information; 3D view; checkCIF report

## Figures and Tables

**Table 1:** Hydrogen-bond geometry (Å, °) *Cg*1 is the centroid of the C41–C46 ring.

*D*—H⋯*A*	*D*—H	H⋯*A*	*D*⋯*A*	*D*—H⋯*A*
N1—H1*A*⋯I2^i^	0.87 (2)	2.85 (3)	3.645 (4)	152 (4)
N1—H1*B*⋯I2	0.84 (2)	2.96 (2)	3.796 (4)	171 (5)
N2—H2⋯I1	0.88 (2)	2.70 (2)	3.563 (4)	168 (4)
C63—H63⋯S1^ii^	0.95	2.95	3.751 (5)	143
C7—H7*A*⋯I1^iii^	0.98	3.02	3.961 (7)	161
C14—H14⋯*Cg*1^iv^	0.95	3.37 (1)	4.08	130

Symmetry codes: (i) 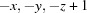; (ii) 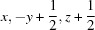; (iii) 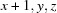; (iv) 
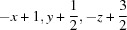
.

**Fig. 1.**
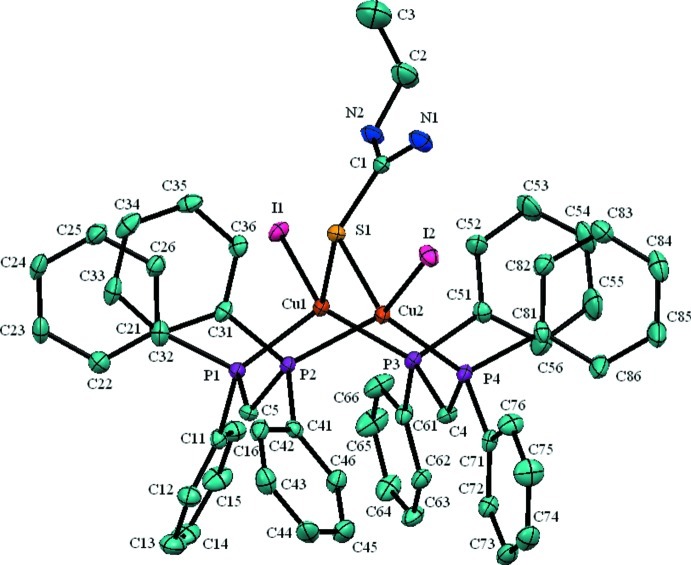
The molecular structure with displacement ellipsoids drawn at the 30% probability level. All hydrogen atoms and solvent molecules are omitted for clarity.

**Fig. 2.**
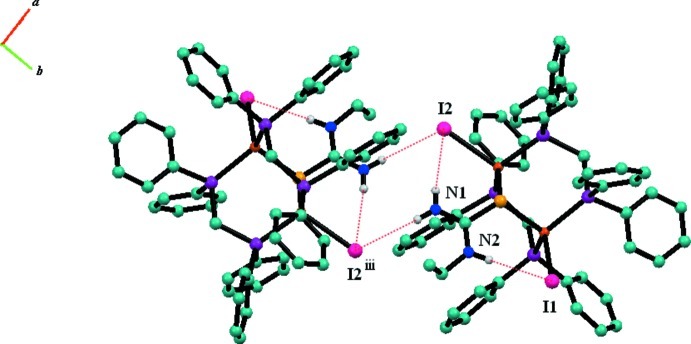
Part of the crystal structure showing intra-intermolecular N—H···I hydrogen bonds shown as red dashed lines.

**Fig. 3.**
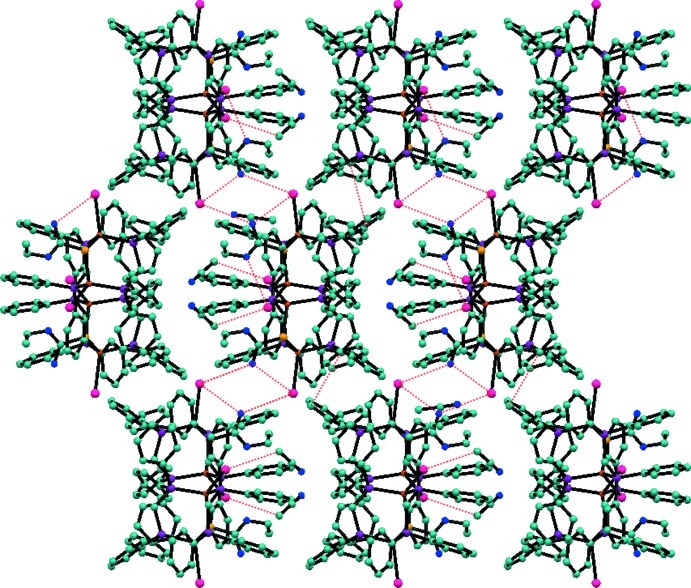
The packing structure of the title complex viewed down the c direction.

## supplementary crystallographic information

### Comment

Metal(I) complexes with mixed phosphine and sulfur donor ligands have attracted much attention in recent years because of various applications based on *e.g.* their luminescent properties (Safin *et al.*, 2010) or antimicrobial activities (Isab *et al.*, 2010). Cu^I^ ions functioning as Lewis acids tend to form covalent bonds with soft ligands and can make close Cu···Cu contacts, features that can promote uncommon bonding, reactivity and catalytic properties of multinuclear copper complexes. Bis(diphenylphosphanyl)methane (dppm), for example, has been reported on multiple occasions to be a diphosphine ligands that is able to lock two metal atoms together in close proximity (Yang *et al.*, 2000; Liaw *et al.*, 2005; Jin *et al.*, 2009). Copper complexes of thiourea ligands such as 1-ethylthiourea (ettu), on the other hand have been of increasing interest due to the variety of their structures and their similarity to metallothioneines, *i.e.* they contain coordinated Cu—S moieties which play an important role in animals and plants. Herein, the crystal structure of a dinuclear copper(I) iodode complex containing both dppm and ettu is described.

The molecular structure of the dinuclear title compound is shown in Fig. 1. Each Cu^I^ ion displays a distorted tetrahedral coordination geometry with internal angles in the range 97.00 (4)–119.56 (4)°. The dppm ligand and the bridging S atom of the ettu ligand force the two copper atoms into close proximity, leading to the formation of a close intramolecular Cu···Cu contact [3.3747 (17) Å]. The Cu—P distances vary from 2.2563 (10) to 2.2786 (11) Å, which are quite similar to those observed in related copper(I) complexes containing the dppm ligand (Yam *et al.*, 2001; Zhou *et al.*, 2001). The Cu—S bond lengths (Cu1—S1 = 2.3450 (11), Cu2—S1 = 2.3493 (11) Å) are shorter than that found in for example [Cu_2_(*µ*-H*L*^1^)_2_(*µ*-dppm)(*η*^1^-dppm)_2_] (H_2_*L*^1^ = 8-mercaptotheophylline), which are 2.392 (5)–2.410 (6) Å (Colacio *et al.*, 1997). The terminal Cu—I bond distances (Cu1—I1 = 2.6694 (6) and Cu2—I2 = 2.6690 (5) Å), are in their typical ranges [see *e.g.* Nimthong *et al.*, 2008; Pakawatchai *et al.*, 2012]. The conformation of the dinuclear complex is such that the two dppm ligands are located on one side of the metal complex, while the two iodine atoms are pointed towards the other side of the complex. This conformation is stabilized by two intramolecular N—H···I hydrogen bonds between the ettu NH_2_ and NHEt moieties and the iodine atoms with N···H distances of 3.796 (4) (for N1···I2) and 3.563 (4) Å (for N2···I1), respectively (Table 1). In the crystal, the N and I atoms are also involved in intramolecular N—H···I hydrogen bonds to form dimers across an inversion center (symmetry code: -*x*, -*y*, -*z* + 1) (Table 1, Fig. 2). The dimers are in turn connected with each other and with the interstitial acetonitrile solvate molecules *via* a range of weaker C—H···I and C—H···S interactions (Table 1) and through a weak C(*sp*^2^)—H···π interaction [C14—H14···*Cg*1^iv^, with H14···*Cg*1^iv^ = 3.38 (3) Å, C14···*Cg*1^iv^ = 4.09 (3) Å and C14—H14···*Cg*1^iv^ = 129.4 (4)°, *Cg*1 = C41—C42—C43—C44—C45—C46 ring, symmetry code: (iv) -*x*+1/2, *y*-1/2, -*z*+1] leading to the formation of a three-dimensional network, Fig. 3 and Table 1. One of the solvent acetonitrile molecules is disordered in a 1:1 ratio across a crystallographic inversion center.

### Experimental

1-Ethylthiourea, ettu, (0.03 g, 0.29 mmol) was dissolved in 30 cm^3^ of acetonitrile at 348 K and then CuI (0.05 g, 0.26 mmol) was added. The mixture was stirred for 4 h and then bis(diphenylphosphanyl)methane, dppm, (0.1 g, 0.26 mmol) was added and the new reaction mixture was heated under reflux for 12 h where upon the precipitate gradually disappeared. The resulting clear solution was filtered off and left to evaporate at room temperature. The crystalline complex, which was deposited upon standing for several days, was filtered off and dried in *vacuo* (0.12 g, yield 67%). Mp = 531–533 K. Analysis found: C 47.77, H 4.02, N 3.68, S 2.26%; calculated for C_112_H_113_Cu_4_IN_7_P_8_S_2_: C 51.09, H 4.29, N 3.72, S 2.43%.

### Refinement

The H atoms were positioned geometrically and refined using a riding model, with C—H = 0.95 with *U*_iso_(H) = 1.2 *U*_eq_(C) for H atoms on C(*sp*^2^) and 0.98–0.99 Å with *U*_iso_(H) = 1.5 *U*_eq_(C) for H atoms on C(*sp*^3^). All H atoms bonded to N atoms were located in a difference Fourier map and refined isotropically, with N—H distances restrained to 0.87 (2) Å with *U*_iso_(H) = 1.2*U*_eq_(N). One of the acetonitrile molecules is disordered in a 1:1 ratio across a crystallographic inversion center and was refined anisotropically.

### Crystal data

**Table:**

[Cu_2_I_2_(C_3_H_8_N_2_S)(C_25_H_22_P_2_)_2_]·1.5C_2_H_3_N	*F*(000) = 2628
*M**_r_* = 1315.37	*D*_x_ = 1.546 Mg m^−^^3^
Monoclinic, *P*2_1_/*c*	Mo *K*α radiation, λ = 0.71073 Å
Hall symbol: -P 2ybc	Cell parameters from 9510 reflections
*a* = 13.7751 (6) Å	θ = 2.3–28.3°
*b* = 24.5147 (11) Å	µ = 2.03 mm^−^^1^
*c* = 18.0172 (8) Å	*T* = 100 K
β = 111.720 (1)°	Hexagon, colorless
*V* = 5652.3 (4) Å^3^	0.28 × 0.17 × 0.10 mm
*Z* = 4	

### Data collection

**Table:**

Bruker SMART CCD diffractometer	10863 independent reflections
Radiation source: fine-focus sealed tube	9108 reflections with *I* > 2σ(*I*)
Graphite monochromator	*R*_int_ = 0.054
phi and ω scans	θ_max_ = 28.3°, θ_min_ = 1.5°
Absorption correction: multi-scan (*SADABS*; Bruker, 2003)	*h* = −18→18
*T*_min_ = 0.670, *T*_max_ = 0.821	*k* = −32→32
53066 measured reflections	*l* = −24→24

### Refinement

**Table:**

Refinement on *F*^2^	Primary atom site location: structure-invariant direct methods
Least-squares matrix: full	Secondary atom site location: difference Fourier map
*R*[*F*^2^ > 2σ(*F*^2^)] = 0.046	Hydrogen site location: inferred from neighbouring sites
*wR*(*F*^2^) = 0.101	H atoms treated by a mixture of independent and constrained refinement
*S* = 1.05	*w* = 1/[σ^2^(*F*_o_^2^) + (0.0416*P*)^2^] where *P* = (*F*_o_^2^ + 2*F*_c_^2^)/3
10863 reflections	(Δ/σ)_max_ = 0.001
643 parameters	Δρ_max_ = 0.84 e Å^−^^3^
3 restraints	Δρ_min_ = −0.55 e Å^−^^3^

### Special details

**Table:**

Geometry. All e.s.d.'s (except the e.s.d. in the dihedral angle between two l.s. planes) are estimated using the full covariance matrix. The cell e.s.d.'s are taken into account individually in the estimation of e.s.d.'s in distances, angles and torsion angles; correlations between e.s.d.'s in cell parameters are only used when they are defined by crystal symmetry. An approximate (isotropic) treatment of cell e.s.d.'s is used for estimating e.s.d.'s involving l.s. planes.
Refinement. Refinement of *F*^2^ against ALL reflections. The weighted *R*-factor *wR* and goodness of fit *S* are based on *F*^2^, conventional *R*-factors *R* are based on *F*, with *F* set to zero for negative *F*^2^. The threshold expression of *F*^2^ > σ(*F*^2^) is used only for calculating *R*-factors(gt) *etc*. and is not relevant to the choice of reflections for refinement. *R*-factors based on *F*^2^ are statistically about twice as large as those based on *F*, and *R*- factors based on ALL data will be even larger.

### Fractional atomic coordinates and isotropic or equivalent isotropic displacement parameters (Å^2^)

**Table:**

	*x*	*y*	*z*	*U*_iso_*/*U*_eq_	Occ. (<1)
C1	0.0596 (3)	0.11641 (17)	0.4430 (2)	0.0175 (8)	
C2	−0.1075 (4)	0.1437 (2)	0.3356 (3)	0.0345 (12)	
H2A	−0.1447	0.1791	0.3243	0.041*	
H2B	−0.1444	0.1196	0.3606	0.041*	
C3	−0.1131 (5)	0.1191 (3)	0.2594 (4)	0.0556 (17)	
H3A	−0.0782	0.1431	0.2334	0.067*	
H3B	−0.1864	0.1144	0.2243	0.067*	
H3C	−0.0782	0.0835	0.2697	0.067*	
C4	0.1919 (3)	0.19363 (16)	0.7423 (2)	0.0156 (8)	
H4A	0.2645	0.2063	0.7704	0.019*	
H4B	0.1533	0.1998	0.7783	0.019*	
C5	0.4659 (3)	0.18775 (15)	0.6705 (2)	0.0145 (8)	
H5A	0.4682	0.1968	0.7246	0.017*	
H5B	0.5376	0.1922	0.6710	0.017*	
C6	0.7716 (5)	0.3044 (3)	0.3672 (5)	0.0482 (16)	
C7	0.8360 (5)	0.3346 (3)	0.4376 (4)	0.0517 (16)	
H7A	0.9098	0.3304	0.4449	0.078*	
H7B	0.8248	0.3204	0.4846	0.078*	
H7C	0.8170	0.3733	0.4306	0.078*	
C8	0.0220 (10)	0.0485 (5)	0.9548 (7)	0.042 (3)	0.50
C9	0.1316 (8)	0.0558 (4)	0.9733 (7)	0.032 (2)	0.50
H9A	0.1710	0.0383	1.0245	0.048*	0.50
H9B	0.1477	0.0949	0.9769	0.048*	0.50
H9C	0.1509	0.0394	0.9312	0.048*	0.50
N4	−0.0633 (9)	0.0414 (6)	0.9455 (8)	0.085 (5)	0.50
C11	0.4225 (3)	0.30180 (16)	0.6545 (2)	0.0158 (8)	
C12	0.5229 (4)	0.31089 (18)	0.7102 (3)	0.0224 (9)	
H12	0.5727	0.2821	0.7243	0.027*	
C13	0.5504 (4)	0.3625 (2)	0.7455 (3)	0.0307 (11)	
H13	0.6185	0.3685	0.7840	0.037*	
C14	0.4789 (5)	0.40422 (19)	0.7242 (3)	0.0321 (12)	
H14	0.4987	0.4396	0.7460	0.039*	
C15	0.3780 (4)	0.39475 (18)	0.6712 (3)	0.0300 (11)	
H15	0.3277	0.4232	0.6585	0.036*	
C16	0.3505 (4)	0.34411 (18)	0.6368 (3)	0.0237 (9)	
H16	0.2811	0.3380	0.6003	0.028*	
C21	0.4350 (3)	0.24310 (16)	0.5205 (2)	0.0156 (8)	
C22	0.5175 (4)	0.27774 (17)	0.5271 (3)	0.0219 (9)	
H22	0.5478	0.2997	0.5734	0.026*	
C23	0.5555 (4)	0.28020 (18)	0.4661 (3)	0.0243 (10)	
H23	0.6100	0.3051	0.4701	0.029*	
C24	0.5159 (4)	0.24728 (19)	0.3998 (3)	0.0228 (9)	
H24	0.5443	0.2485	0.3591	0.027*	
C25	0.4335 (4)	0.21213 (19)	0.3930 (3)	0.0236 (10)	
H25	0.4051	0.1893	0.3475	0.028*	
C26	0.3930 (4)	0.21056 (17)	0.4533 (3)	0.0197 (9)	
H26	0.3362	0.1870	0.4483	0.024*	
C31	0.4709 (3)	0.09683 (16)	0.5689 (2)	0.0172 (8)	
C32	0.5688 (4)	0.11185 (18)	0.5680 (3)	0.0220 (9)	
H32	0.6162	0.1320	0.6113	0.026*	
C33	0.5963 (4)	0.09741 (19)	0.5043 (3)	0.0256 (10)	
H33	0.6629	0.1073	0.5041	0.031*	
C34	0.5263 (4)	0.06833 (18)	0.4403 (3)	0.0240 (10)	
H34	0.5450	0.0585	0.3964	0.029*	
C35	0.4305 (4)	0.05394 (18)	0.4409 (3)	0.0221 (9)	
H35	0.3824	0.0348	0.3967	0.027*	
C36	0.4028 (3)	0.06715 (17)	0.5057 (3)	0.0179 (8)	
H36	0.3372	0.0558	0.5065	0.022*	
C41	0.5279 (3)	0.08573 (16)	0.7403 (2)	0.0156 (8)	
C42	0.6097 (4)	0.05246 (17)	0.7397 (3)	0.0200 (9)	
H42	0.6153	0.0433	0.6902	0.024*	
C43	0.6820 (4)	0.03278 (19)	0.8094 (3)	0.0253 (10)	
H43	0.7378	0.0106	0.8080	0.030*	
C44	0.6741 (4)	0.04503 (19)	0.8823 (3)	0.0259 (10)	
H44	0.7247	0.0317	0.9306	0.031*	
C45	0.5915 (4)	0.07692 (19)	0.8837 (3)	0.0264 (10)	
H45	0.5842	0.0846	0.9330	0.032*	
C46	0.5206 (4)	0.09737 (18)	0.8140 (3)	0.0213 (9)	
H46	0.4654	0.1199	0.8158	0.026*	
C51	−0.0082 (3)	0.22244 (16)	0.6224 (3)	0.0168 (8)	
C52	−0.0682 (4)	0.20973 (19)	0.5442 (3)	0.0243 (10)	
H52	−0.0361	0.2066	0.5060	0.029*	
C53	−0.1756 (4)	0.2015 (2)	0.5208 (3)	0.0332 (11)	
H53	−0.2159	0.1917	0.4671	0.040*	
C54	−0.2232 (4)	0.20746 (19)	0.5747 (3)	0.0300 (11)	
H54	−0.2967	0.2029	0.5580	0.036*	
C55	−0.1650 (4)	0.2200 (2)	0.6533 (4)	0.0392 (14)	
H55	−0.1984	0.2240	0.6906	0.047*	
C56	−0.0563 (4)	0.2270 (2)	0.6783 (3)	0.0348 (12)	
H56	−0.0158	0.2347	0.7326	0.042*	
C61	0.1374 (3)	0.30465 (16)	0.6903 (3)	0.0171 (8)	
C62	0.1786 (3)	0.31816 (17)	0.7703 (3)	0.0183 (8)	
H62	0.2064	0.2904	0.8093	0.022*	
C63	0.1796 (4)	0.37259 (18)	0.7944 (3)	0.0245 (10)	
H63	0.2093	0.3816	0.8496	0.029*	
C64	0.1379 (4)	0.41329 (18)	0.7387 (3)	0.0262 (10)	
H64	0.1385	0.4501	0.7553	0.031*	
C65	0.0951 (5)	0.39985 (19)	0.6582 (3)	0.0330 (12)	
H65	0.0655	0.4275	0.6195	0.040*	
C66	0.0955 (4)	0.34600 (19)	0.6340 (3)	0.0288 (11)	
H66	0.0671	0.3372	0.5787	0.035*	
C71	0.2684 (3)	0.09250 (16)	0.8223 (2)	0.0159 (8)	
C72	0.3174 (3)	0.12398 (17)	0.8905 (2)	0.0189 (9)	
H72	0.3117	0.1626	0.8877	0.023*	
C73	0.3747 (4)	0.09874 (19)	0.9631 (3)	0.0233 (9)	
H73	0.4077	0.1206	1.0092	0.028*	
C74	0.3843 (4)	0.04333 (19)	0.9690 (3)	0.0246 (10)	
H74	0.4231	0.0266	1.0188	0.030*	
C75	0.3364 (4)	0.01183 (18)	0.9014 (3)	0.0300 (11)	
H75	0.3436	−0.0267	0.9049	0.036*	
C76	0.2784 (4)	0.03575 (17)	0.8289 (3)	0.0226 (9)	
H76	0.2450	0.0135	0.7834	0.027*	
C81	0.0611 (3)	0.09752 (16)	0.7085 (3)	0.0163 (8)	
C82	−0.0038 (4)	0.07981 (18)	0.6340 (3)	0.0230 (9)	
H82	0.0212	0.0787	0.5915	0.028*	
C83	−0.1055 (4)	0.0635 (2)	0.6206 (3)	0.0265 (10)	
H83	−0.1497	0.0513	0.5691	0.032*	
C84	−0.1423 (4)	0.06518 (19)	0.6821 (3)	0.0286 (11)	
H84	−0.2120	0.0544	0.6730	0.034*	
C85	−0.0776 (4)	0.0824 (2)	0.7568 (3)	0.0378 (14)	
H85	−0.1029	0.0836	0.7991	0.045*	
C86	0.0249 (4)	0.0982 (2)	0.7706 (3)	0.0339 (13)	
H86	0.0697	0.1094	0.8225	0.041*	
N1	0.0293 (3)	0.06564 (16)	0.4455 (2)	0.0234 (8)	
N2	−0.0010 (3)	0.15260 (15)	0.3924 (2)	0.0218 (8)	
N3	0.7233 (5)	0.2815 (3)	0.3116 (4)	0.0662 (18)	
P1	0.38018 (8)	0.23788 (4)	0.59951 (6)	0.0136 (2)	
P2	0.42899 (8)	0.11516 (4)	0.65079 (6)	0.0130 (2)	
P3	0.13118 (8)	0.23514 (4)	0.65131 (6)	0.0130 (2)	
P4	0.19412 (8)	0.12008 (4)	0.72331 (6)	0.0132 (2)	
S1	0.18473 (8)	0.13532 (4)	0.50372 (6)	0.01496 (19)	
Cu1	0.20473 (4)	0.223317 (19)	0.55840 (3)	0.01406 (11)	
Cu2	0.25996 (4)	0.095310 (19)	0.63076 (3)	0.01382 (11)	
I1	0.10743 (2)	0.286304 (11)	0.431721 (16)	0.01865 (7)	
I2	0.23544 (2)	−0.012447 (10)	0.611802 (17)	0.01886 (7)	
H1A	−0.032 (2)	0.0543 (19)	0.414 (2)	0.023*	
H1B	0.073 (3)	0.0450 (17)	0.479 (2)	0.023*	
H2	0.024 (4)	0.1856 (11)	0.394 (3)	0.023*	

### Atomic displacement parameters (Å^2^)

**Table:**

	*U*^11^	*U*^22^	*U*^33^	*U*^12^	*U*^13^	*U*^23^
C1	0.016 (2)	0.024 (2)	0.014 (2)	−0.0039 (16)	0.0078 (18)	−0.0042 (16)
C2	0.022 (3)	0.044 (3)	0.028 (3)	−0.005 (2)	−0.001 (2)	0.008 (2)
C3	0.042 (4)	0.077 (5)	0.040 (4)	−0.007 (3)	0.006 (3)	−0.002 (3)
C4	0.018 (2)	0.0165 (18)	0.0123 (19)	0.0002 (16)	0.0052 (17)	−0.0003 (15)
C5	0.0133 (19)	0.0162 (18)	0.0129 (19)	−0.0033 (15)	0.0036 (17)	−0.0029 (15)
C6	0.028 (3)	0.063 (4)	0.058 (4)	0.011 (3)	0.022 (3)	0.017 (4)
C7	0.044 (4)	0.049 (4)	0.058 (4)	0.022 (3)	0.014 (3)	0.002 (3)
C8	0.050 (7)	0.042 (6)	0.044 (7)	0.000 (5)	0.029 (6)	−0.014 (5)
C9	0.030 (5)	0.026 (5)	0.041 (6)	0.017 (4)	0.016 (5)	0.020 (4)
N4	0.039 (7)	0.148 (13)	0.081 (9)	−0.055 (8)	0.038 (7)	−0.071 (9)
C11	0.020 (2)	0.0127 (17)	0.017 (2)	−0.0039 (16)	0.0096 (18)	0.0000 (15)
C12	0.024 (2)	0.021 (2)	0.023 (2)	−0.0022 (18)	0.009 (2)	−0.0027 (17)
C13	0.038 (3)	0.029 (3)	0.023 (2)	−0.012 (2)	0.008 (2)	−0.006 (2)
C14	0.054 (4)	0.018 (2)	0.026 (3)	−0.009 (2)	0.017 (3)	−0.0102 (19)
C15	0.045 (3)	0.017 (2)	0.028 (3)	0.003 (2)	0.015 (3)	−0.0036 (19)
C16	0.028 (2)	0.022 (2)	0.020 (2)	−0.0013 (18)	0.007 (2)	−0.0024 (17)
C21	0.0149 (19)	0.0188 (19)	0.016 (2)	0.0000 (16)	0.0091 (18)	0.0010 (16)
C22	0.021 (2)	0.022 (2)	0.021 (2)	−0.0024 (17)	0.007 (2)	−0.0010 (17)
C23	0.019 (2)	0.030 (2)	0.028 (3)	0.0000 (18)	0.013 (2)	0.0094 (19)
C24	0.022 (2)	0.031 (2)	0.020 (2)	0.0058 (19)	0.013 (2)	0.0085 (18)
C25	0.023 (2)	0.032 (2)	0.018 (2)	0.0013 (19)	0.010 (2)	−0.0020 (18)
C26	0.023 (2)	0.021 (2)	0.017 (2)	−0.0019 (17)	0.009 (2)	0.0020 (16)
C31	0.020 (2)	0.0180 (19)	0.017 (2)	0.0019 (16)	0.0103 (19)	−0.0006 (16)
C32	0.022 (2)	0.023 (2)	0.025 (2)	−0.0018 (17)	0.014 (2)	0.0014 (18)
C33	0.025 (2)	0.030 (2)	0.029 (3)	0.0041 (19)	0.019 (2)	0.006 (2)
C34	0.030 (3)	0.029 (2)	0.019 (2)	0.013 (2)	0.015 (2)	0.0040 (18)
C35	0.024 (2)	0.026 (2)	0.015 (2)	0.0069 (18)	0.0054 (19)	−0.0045 (17)
C36	0.015 (2)	0.021 (2)	0.020 (2)	0.0043 (16)	0.0093 (19)	−0.0015 (17)
C41	0.015 (2)	0.0137 (18)	0.017 (2)	−0.0038 (15)	0.0047 (18)	−0.0006 (15)
C42	0.024 (2)	0.020 (2)	0.020 (2)	−0.0036 (17)	0.013 (2)	−0.0036 (17)
C43	0.018 (2)	0.027 (2)	0.032 (3)	−0.0002 (18)	0.010 (2)	0.005 (2)
C44	0.021 (2)	0.033 (3)	0.018 (2)	−0.0037 (19)	0.001 (2)	0.0081 (19)
C45	0.030 (3)	0.030 (2)	0.018 (2)	−0.005 (2)	0.008 (2)	0.0009 (19)
C46	0.021 (2)	0.024 (2)	0.021 (2)	0.0013 (18)	0.010 (2)	0.0021 (17)
C51	0.013 (2)	0.0173 (19)	0.019 (2)	0.0024 (15)	0.0053 (18)	0.0031 (16)
C52	0.019 (2)	0.035 (3)	0.018 (2)	−0.0011 (19)	0.005 (2)	0.0028 (19)
C53	0.014 (2)	0.046 (3)	0.033 (3)	−0.004 (2)	0.001 (2)	0.004 (2)
C54	0.016 (2)	0.031 (3)	0.042 (3)	0.0027 (19)	0.010 (2)	0.012 (2)
C55	0.022 (3)	0.059 (4)	0.044 (4)	0.000 (2)	0.020 (3)	0.003 (3)
C56	0.028 (3)	0.054 (3)	0.029 (3)	−0.004 (2)	0.018 (3)	−0.008 (2)
C61	0.022 (2)	0.0138 (18)	0.020 (2)	−0.0007 (16)	0.0127 (19)	0.0000 (16)
C62	0.023 (2)	0.0181 (19)	0.016 (2)	0.0002 (16)	0.0093 (19)	0.0021 (16)
C63	0.036 (3)	0.022 (2)	0.019 (2)	−0.0058 (19)	0.014 (2)	−0.0056 (17)
C64	0.040 (3)	0.016 (2)	0.028 (3)	0.0005 (19)	0.019 (2)	−0.0014 (18)
C65	0.055 (4)	0.018 (2)	0.028 (3)	0.010 (2)	0.017 (3)	0.0045 (19)
C66	0.044 (3)	0.025 (2)	0.017 (2)	0.007 (2)	0.010 (2)	0.0011 (18)
C71	0.019 (2)	0.0151 (18)	0.018 (2)	0.0009 (15)	0.0109 (18)	0.0031 (15)
C72	0.023 (2)	0.0176 (19)	0.019 (2)	0.0007 (17)	0.012 (2)	0.0007 (16)
C73	0.023 (2)	0.032 (2)	0.015 (2)	0.0041 (19)	0.008 (2)	0.0042 (18)
C74	0.023 (2)	0.031 (2)	0.020 (2)	0.0088 (19)	0.007 (2)	0.0080 (19)
C75	0.039 (3)	0.016 (2)	0.032 (3)	0.005 (2)	0.010 (3)	0.0057 (19)
C76	0.031 (3)	0.0145 (19)	0.021 (2)	0.0004 (18)	0.008 (2)	−0.0010 (17)
C81	0.017 (2)	0.0123 (18)	0.023 (2)	−0.0026 (15)	0.0116 (19)	−0.0014 (15)
C82	0.022 (2)	0.029 (2)	0.020 (2)	−0.0004 (18)	0.011 (2)	−0.0019 (18)
C83	0.019 (2)	0.032 (2)	0.027 (3)	−0.0050 (19)	0.006 (2)	−0.005 (2)
C84	0.024 (2)	0.023 (2)	0.046 (3)	−0.0044 (19)	0.021 (2)	−0.003 (2)
C85	0.037 (3)	0.049 (3)	0.039 (3)	−0.022 (3)	0.027 (3)	−0.019 (3)
C86	0.039 (3)	0.043 (3)	0.031 (3)	−0.021 (2)	0.026 (3)	−0.018 (2)
N1	0.0152 (19)	0.024 (2)	0.024 (2)	−0.0025 (16)	−0.0004 (17)	0.0010 (16)
N2	0.0161 (18)	0.0247 (19)	0.0180 (19)	−0.0041 (15)	−0.0014 (16)	0.0031 (15)
N3	0.040 (3)	0.101 (5)	0.053 (4)	−0.004 (3)	0.012 (3)	0.002 (4)
P1	0.0149 (5)	0.0143 (5)	0.0130 (5)	−0.0025 (4)	0.0067 (4)	−0.0017 (4)
P2	0.0130 (5)	0.0146 (5)	0.0132 (5)	−0.0014 (4)	0.0071 (4)	−0.0021 (4)
P3	0.0143 (5)	0.0143 (5)	0.0117 (5)	0.0007 (4)	0.0064 (4)	0.0003 (4)
P4	0.0158 (5)	0.0129 (5)	0.0134 (5)	−0.0008 (4)	0.0084 (4)	−0.0002 (4)
S1	0.0141 (5)	0.0169 (5)	0.0137 (5)	−0.0016 (4)	0.0051 (4)	−0.0014 (4)
Cu1	0.0151 (2)	0.0159 (2)	0.0129 (2)	−0.00091 (19)	0.0072 (2)	−0.00045 (18)
Cu2	0.0135 (2)	0.0153 (2)	0.0147 (2)	−0.00164 (18)	0.0076 (2)	−0.00205 (18)
I1	0.02440 (15)	0.01919 (13)	0.01354 (14)	0.00084 (10)	0.00841 (12)	0.00374 (10)
I2	0.01749 (14)	0.01558 (13)	0.02425 (15)	−0.00252 (10)	0.00860 (12)	−0.00355 (10)

### Geometric parameters (Å, º)

**Table:**

C1—N1	1.319 (5)	C42—H42	0.9500
C1—N2	1.323 (6)	C43—C44	1.389 (6)
C1—S1	1.729 (4)	C43—H43	0.9500
C2—N2	1.462 (6)	C44—C45	1.388 (7)
C2—C3	1.476 (8)	C44—H44	0.9500
C2—H2A	0.9900	C45—C46	1.369 (6)
C2—H2B	0.9900	C45—H45	0.9500
C3—H3A	0.9800	C46—H46	0.9500
C3—H3B	0.9800	C51—C52	1.380 (6)
C3—H3C	0.9800	C51—C56	1.399 (6)
C4—P4	1.838 (4)	C51—P3	1.821 (4)
C4—P3	1.846 (4)	C52—C53	1.395 (7)
C4—H4A	0.9900	C52—H52	0.9500
C4—H4B	0.9900	C53—C54	1.365 (7)
C5—P1	1.850 (4)	C53—H53	0.9500
C5—P2	1.849 (4)	C54—C55	1.380 (8)
C5—H5A	0.9900	C54—H54	0.9500
C5—H5B	0.9900	C55—C56	1.404 (7)
C6—N3	1.125 (9)	C55—H55	0.9500
C6—C7	1.453 (10)	C56—H56	0.9500
C7—H7A	0.9800	C61—C62	1.380 (6)
C7—H7B	0.9800	C61—C66	1.399 (6)
C7—H7C	0.9800	C61—P3	1.833 (4)
C8—N4	1.138 (16)	C62—C63	1.402 (6)
C8—C9	1.431 (16)	C62—H62	0.9500
C9—H9A	0.9800	C63—C64	1.381 (7)
C9—H9B	0.9800	C63—H63	0.9500
C9—H9C	0.9800	C64—C65	1.388 (7)
C11—C16	1.389 (6)	C64—H64	0.9500
C11—C12	1.393 (6)	C65—C66	1.391 (6)
C11—P1	1.830 (4)	C65—H65	0.9500
C12—C13	1.403 (6)	C66—H66	0.9500
C12—H12	0.9500	C71—C72	1.396 (6)
C13—C14	1.373 (8)	C71—C76	1.399 (5)
C13—H13	0.9500	C71—P4	1.826 (4)
C14—C15	1.384 (8)	C72—C73	1.397 (6)
C14—H14	0.9500	C72—H72	0.9500
C15—C16	1.377 (6)	C73—C74	1.365 (6)
C15—H15	0.9500	C73—H73	0.9500
C16—H16	0.9500	C74—C75	1.386 (7)
C21—C26	1.385 (6)	C74—H74	0.9500
C21—C22	1.388 (6)	C75—C76	1.384 (7)
C21—P1	1.847 (4)	C75—H75	0.9500
C22—C23	1.382 (6)	C76—H76	0.9500
C22—H22	0.9500	C81—C82	1.378 (6)
C23—C24	1.376 (7)	C81—C86	1.384 (6)
C23—H23	0.9500	C81—P4	1.837 (4)
C24—C25	1.394 (6)	C82—C83	1.390 (6)
C24—H24	0.9500	C82—H82	0.9500
C25—C26	1.392 (6)	C83—C84	1.379 (6)
C25—H25	0.9500	C83—H83	0.9500
C26—H26	0.9500	C84—C85	1.377 (7)
C31—C36	1.384 (6)	C84—H84	0.9500
C31—C32	1.404 (6)	C85—C86	1.394 (7)
C31—P2	1.828 (4)	C85—H85	0.9500
C32—C33	1.381 (6)	C86—H86	0.9500
C32—H32	0.9500	N1—H1A	0.873 (19)
C33—C34	1.395 (7)	N1—H1B	0.841 (19)
C33—H33	0.9500	N2—H2	0.875 (19)
C34—C35	1.370 (7)	P1—Cu1	2.2786 (11)
C34—H34	0.9500	P2—Cu2	2.2730 (11)
C35—C36	1.394 (5)	P3—Cu1	2.2733 (10)
C35—H35	0.9500	P4—Cu2	2.2563 (10)
C36—H36	0.9500	S1—Cu1	2.3450 (11)
C41—C42	1.394 (6)	S1—Cu2	2.3493 (11)
C41—C46	1.397 (6)	Cu1—I1	2.6694 (6)
C41—P2	1.831 (4)	Cu2—I2	2.6690 (5)
C42—C43	1.370 (7)		
			
N1—C1—N2	122.0 (4)	C56—C51—P3	120.7 (4)
N1—C1—S1	119.0 (3)	C51—C52—C53	120.6 (4)
N2—C1—S1	118.9 (3)	C51—C52—H52	119.7
N2—C2—C3	114.0 (5)	C53—C52—H52	119.7
N2—C2—H2A	108.8	C54—C53—C52	120.2 (5)
C3—C2—H2A	108.8	C54—C53—H53	119.9
N2—C2—H2B	108.8	C52—C53—H53	119.9
C3—C2—H2B	108.8	C53—C54—C55	120.3 (5)
H2A—C2—H2B	107.7	C53—C54—H54	119.8
C2—C3—H3A	109.5	C55—C54—H54	119.8
C2—C3—H3B	109.5	C54—C55—C56	120.1 (5)
H3A—C3—H3B	109.5	C54—C55—H55	120.0
C2—C3—H3C	109.5	C56—C55—H55	120.0
H3A—C3—H3C	109.5	C51—C56—C55	119.5 (5)
H3B—C3—H3C	109.5	C51—C56—H56	120.2
P4—C4—P3	114.1 (2)	C55—C56—H56	120.2
P4—C4—H4A	108.7	C62—C61—C66	118.9 (4)
P3—C4—H4A	108.7	C62—C61—P3	124.5 (3)
P4—C4—H4B	108.7	C66—C61—P3	116.6 (3)
P3—C4—H4B	108.7	C61—C62—C63	120.3 (4)
H4A—C4—H4B	107.6	C61—C62—H62	119.8
P1—C5—P2	116.5 (2)	C63—C62—H62	119.8
P1—C5—H5A	108.2	C64—C63—C62	120.6 (4)
P2—C5—H5A	108.2	C64—C63—H63	119.7
P1—C5—H5B	108.2	C62—C63—H63	119.7
P2—C5—H5B	108.2	C63—C64—C65	119.3 (4)
H5A—C5—H5B	107.3	C63—C64—H64	120.3
N3—C6—C7	178.1 (7)	C65—C64—H64	120.3
C6—C7—H7A	109.5	C66—C65—C64	120.2 (4)
C6—C7—H7B	109.5	C66—C65—H65	119.9
H7A—C7—H7B	109.5	C64—C65—H65	119.9
C6—C7—H7C	109.5	C65—C66—C61	120.6 (4)
H7A—C7—H7C	109.5	C65—C66—H66	119.7
H7B—C7—H7C	109.5	C61—C66—H66	119.7
N4—C8—C9	175.2 (15)	C72—C71—C76	118.3 (4)
C8—C9—H9A	109.5	C72—C71—P4	124.7 (3)
C8—C9—H9B	109.5	C76—C71—P4	117.0 (3)
H9A—C9—H9B	109.5	C73—C72—C71	120.1 (4)
C8—C9—H9C	109.5	C73—C72—H72	120.0
H9A—C9—H9C	109.5	C71—C72—H72	120.0
H9B—C9—H9C	109.5	C74—C73—C72	121.3 (4)
C16—C11—C12	118.7 (4)	C74—C73—H73	119.4
C16—C11—P1	117.2 (3)	C72—C73—H73	119.4
C12—C11—P1	124.0 (3)	C73—C74—C75	119.0 (4)
C11—C12—C13	120.0 (4)	C73—C74—H74	120.5
C11—C12—H12	120.0	C75—C74—H74	120.5
C13—C12—H12	120.0	C76—C75—C74	121.0 (4)
C14—C13—C12	120.0 (5)	C76—C75—H75	119.5
C14—C13—H13	120.0	C74—C75—H75	119.5
C12—C13—H13	120.0	C75—C76—C71	120.4 (4)
C13—C14—C15	120.0 (4)	C75—C76—H76	119.8
C13—C14—H14	120.0	C71—C76—H76	119.8
C15—C14—H14	120.0	C82—C81—C86	119.6 (4)
C14—C15—C16	120.1 (5)	C82—C81—P4	119.3 (3)
C14—C15—H15	120.0	C86—C81—P4	121.1 (4)
C16—C15—H15	120.0	C81—C82—C83	120.6 (4)
C15—C16—C11	121.1 (5)	C81—C82—H82	119.7
C15—C16—H16	119.5	C83—C82—H82	119.7
C11—C16—H16	119.5	C84—C83—C82	119.9 (5)
C26—C21—C22	119.5 (4)	C84—C83—H83	120.0
C26—C21—P1	118.4 (3)	C82—C83—H83	120.0
C22—C21—P1	122.1 (3)	C83—C84—C85	119.8 (4)
C23—C22—C21	119.8 (4)	C83—C84—H84	120.1
C23—C22—H22	120.1	C85—C84—H84	120.1
C21—C22—H22	120.1	C84—C85—C86	120.5 (4)
C24—C23—C22	121.3 (4)	C84—C85—H85	119.8
C24—C23—H23	119.3	C86—C85—H85	119.8
C22—C23—H23	119.3	C81—C86—C85	119.7 (5)
C23—C24—C25	119.1 (4)	C81—C86—H86	120.2
C23—C24—H24	120.4	C85—C86—H86	120.2
C25—C24—H24	120.4	C1—N1—H1A	122 (3)
C26—C25—C24	119.8 (4)	C1—N1—H1B	116 (4)
C26—C25—H25	120.1	H1A—N1—H1B	122 (5)
C24—C25—H25	120.1	C1—N2—C2	126.6 (4)
C21—C26—C25	120.4 (4)	C1—N2—H2	117 (3)
C21—C26—H26	119.8	C2—N2—H2	116 (3)
C25—C26—H26	119.8	C11—P1—C21	102.63 (18)
C36—C31—C32	119.3 (4)	C11—P1—C5	101.56 (19)
C36—C31—P2	118.2 (3)	C21—P1—C5	103.90 (18)
C32—C31—P2	122.5 (3)	C11—P1—Cu1	113.42 (14)
C33—C32—C31	120.2 (4)	C21—P1—Cu1	116.59 (14)
C33—C32—H32	119.9	C5—P1—Cu1	116.72 (13)
C31—C32—H32	119.9	C31—P2—C41	105.06 (19)
C32—C33—C34	119.9 (4)	C31—P2—C5	104.17 (18)
C32—C33—H33	120.0	C41—P2—C5	98.51 (18)
C34—C33—H33	120.0	C31—P2—Cu2	115.48 (15)
C35—C34—C33	119.9 (4)	C41—P2—Cu2	116.10 (13)
C35—C34—H34	120.0	C5—P2—Cu2	115.44 (13)
C33—C34—H34	120.0	C51—P3—C61	99.41 (19)
C34—C35—C36	120.7 (4)	C51—P3—C4	104.06 (19)
C34—C35—H35	119.7	C61—P3—C4	103.32 (19)
C36—C35—H35	119.7	C51—P3—Cu1	118.56 (14)
C31—C36—C35	119.9 (4)	C61—P3—Cu1	115.48 (12)
C31—C36—H36	120.0	C4—P3—Cu1	113.87 (13)
C35—C36—H36	120.0	C71—P4—C81	101.49 (19)
C42—C41—C46	118.0 (4)	C71—P4—C4	102.91 (18)
C42—C41—P2	124.3 (3)	C81—P4—C4	103.77 (18)
C46—C41—P2	117.7 (3)	C71—P4—Cu2	112.88 (13)
C43—C42—C41	120.9 (4)	C81—P4—Cu2	117.63 (13)
C43—C42—H42	119.5	C4—P4—Cu2	116.13 (12)
C41—C42—H42	119.5	C1—S1—Cu1	116.57 (15)
C42—C43—C44	120.4 (4)	C1—S1—Cu2	118.81 (14)
C42—C43—H43	119.8	Cu1—S1—Cu2	91.94 (4)
C44—C43—H43	119.8	P3—Cu1—P1	116.67 (4)
C45—C44—C43	119.3 (4)	P3—Cu1—S1	114.30 (4)
C45—C44—H44	120.3	P1—Cu1—S1	103.08 (4)
C43—C44—H44	120.3	P3—Cu1—I1	109.65 (3)
C46—C45—C44	120.1 (4)	P1—Cu1—I1	108.89 (3)
C46—C45—H45	120.0	S1—Cu1—I1	103.25 (3)
C44—C45—H45	120.0	P4—Cu2—P2	119.56 (4)
C45—C46—C41	121.2 (4)	P4—Cu2—S1	117.04 (4)
C45—C46—H46	119.4	P2—Cu2—S1	97.00 (4)
C41—C46—H46	119.4	P4—Cu2—I2	107.13 (3)
C52—C51—C56	119.2 (4)	P2—Cu2—I2	107.81 (3)
C52—C51—P3	120.0 (3)	S1—Cu2—I2	107.41 (3)
			
C16—C11—C12—C13	−1.9 (6)	C42—C41—P2—C5	−112.7 (4)
P1—C11—C12—C13	175.6 (3)	C46—C41—P2—C5	67.4 (3)
C11—C12—C13—C14	−0.9 (7)	C42—C41—P2—Cu2	123.4 (3)
C12—C13—C14—C15	3.4 (7)	C46—C41—P2—Cu2	−56.4 (4)
C13—C14—C15—C16	−3.0 (7)	P1—C5—P2—C31	77.2 (2)
C14—C15—C16—C11	0.2 (7)	P1—C5—P2—C41	−174.8 (2)
C12—C11—C16—C15	2.2 (6)	P1—C5—P2—Cu2	−50.5 (2)
P1—C11—C16—C15	−175.4 (3)	C52—C51—P3—C61	121.4 (4)
C26—C21—C22—C23	1.3 (7)	C56—C51—P3—C61	−56.7 (4)
P1—C21—C22—C23	−179.8 (3)	C52—C51—P3—C4	−132.2 (4)
C21—C22—C23—C24	−2.6 (7)	C56—C51—P3—C4	49.7 (4)
C22—C23—C24—C25	2.2 (7)	C52—C51—P3—Cu1	−4.5 (4)
C23—C24—C25—C26	−0.3 (7)	C56—C51—P3—Cu1	177.4 (4)
C22—C21—C26—C25	0.5 (7)	C62—C61—P3—C51	106.4 (4)
P1—C21—C26—C25	−178.4 (3)	C66—C61—P3—C51	−72.5 (4)
C24—C25—C26—C21	−1.0 (7)	C62—C61—P3—C4	−0.6 (4)
C36—C31—C32—C33	0.4 (7)	C66—C61—P3—C4	−179.5 (3)
P2—C31—C32—C33	−179.4 (3)	C62—C61—P3—Cu1	−125.6 (3)
C31—C32—C33—C34	0.7 (7)	C66—C61—P3—Cu1	55.5 (4)
C32—C33—C34—C35	−0.3 (7)	P4—C4—P3—C51	74.8 (2)
C33—C34—C35—C36	−1.3 (7)	P4—C4—P3—C61	178.3 (2)
C32—C31—C36—C35	−2.0 (6)	P4—C4—P3—Cu1	−55.7 (2)
P2—C31—C36—C35	177.9 (3)	C72—C71—P4—C81	−112.8 (4)
C34—C35—C36—C31	2.4 (7)	C76—C71—P4—C81	68.8 (3)
C46—C41—C42—C43	−1.4 (6)	C72—C71—P4—C4	−5.6 (4)
P2—C41—C42—C43	178.7 (3)	C76—C71—P4—C4	176.0 (3)
C41—C42—C43—C44	1.0 (7)	C72—C71—P4—Cu2	120.4 (3)
C42—C43—C44—C45	0.7 (7)	C76—C71—P4—Cu2	−58.1 (3)
C43—C44—C45—C46	−2.0 (7)	C82—C81—P4—C71	−141.0 (3)
C44—C45—C46—C41	1.6 (7)	C86—C81—P4—C71	39.3 (4)
C42—C41—C46—C45	0.1 (6)	C82—C81—P4—C4	112.5 (4)
P2—C41—C46—C45	−180.0 (3)	C86—C81—P4—C4	−67.2 (4)
C56—C51—C52—C53	0.0 (7)	C82—C81—P4—Cu2	−17.3 (4)
P3—C51—C52—C53	−178.1 (4)	C86—C81—P4—Cu2	163.0 (4)
C51—C52—C53—C54	1.9 (8)	P3—C4—P4—C71	175.0 (2)
C52—C53—C54—C55	−2.0 (8)	P3—C4—P4—C81	−79.6 (2)
C53—C54—C55—C56	0.2 (8)	P3—C4—P4—Cu2	51.1 (3)
C52—C51—C56—C55	−1.8 (8)	N1—C1—S1—Cu1	−143.2 (3)
P3—C51—C56—C55	176.3 (4)	N2—C1—S1—Cu1	40.2 (4)
C54—C55—C56—C51	1.7 (8)	N1—C1—S1—Cu2	−34.7 (4)
C66—C61—C62—C63	−0.9 (6)	N2—C1—S1—Cu2	148.8 (3)
P3—C61—C62—C63	−179.8 (3)	C51—P3—Cu1—P1	−178.53 (15)
C61—C62—C63—C64	1.1 (7)	C61—P3—Cu1—P1	63.72 (17)
C62—C63—C64—C65	−0.3 (7)	C4—P3—Cu1—P1	−55.62 (15)
C63—C64—C65—C66	−0.8 (8)	C51—P3—Cu1—S1	−58.22 (16)
C64—C65—C66—C61	1.0 (8)	C61—P3—Cu1—S1	−175.97 (16)
C62—C61—C66—C65	−0.2 (7)	C4—P3—Cu1—S1	64.68 (15)
P3—C61—C66—C65	178.8 (4)	C51—P3—Cu1—I1	57.13 (15)
C76—C71—C72—C73	0.1 (6)	C61—P3—Cu1—I1	−60.62 (17)
P4—C71—C72—C73	−178.3 (3)	C4—P3—Cu1—I1	−179.96 (14)
C71—C72—C73—C74	0.0 (6)	C11—P1—Cu1—P3	−48.21 (15)
C72—C73—C74—C75	0.4 (7)	C21—P1—Cu1—P3	−167.11 (15)
C73—C74—C75—C76	−1.0 (7)	C5—P1—Cu1—P3	69.35 (14)
C74—C75—C76—C71	1.1 (7)	C11—P1—Cu1—S1	−174.32 (14)
C72—C71—C76—C75	−0.7 (6)	C21—P1—Cu1—S1	66.78 (15)
P4—C71—C76—C75	177.8 (4)	C5—P1—Cu1—S1	−56.77 (14)
C86—C81—C82—C83	1.0 (7)	C11—P1—Cu1—I1	76.52 (14)
P4—C81—C82—C83	−178.7 (4)	C21—P1—Cu1—I1	−42.38 (15)
C81—C82—C83—C84	0.1 (7)	C5—P1—Cu1—I1	−165.93 (14)
C82—C83—C84—C85	−0.6 (8)	C1—S1—Cu1—P3	68.75 (16)
C83—C84—C85—C86	0.0 (8)	Cu2—S1—Cu1—P3	−55.03 (5)
C82—C81—C86—C85	−1.7 (8)	C1—S1—Cu1—P1	−163.63 (15)
P4—C81—C86—C85	178.0 (4)	Cu2—S1—Cu1—P1	72.59 (4)
C84—C85—C86—C81	1.2 (9)	C1—S1—Cu1—I1	−50.29 (15)
N1—C1—N2—C2	2.2 (7)	Cu2—S1—Cu1—I1	−174.07 (3)
S1—C1—N2—C2	178.6 (4)	C71—P4—Cu2—P2	−56.81 (15)
C3—C2—N2—C1	−84.3 (6)	C81—P4—Cu2—P2	−174.52 (15)
C16—C11—P1—C21	98.8 (3)	C4—P4—Cu2—P2	61.69 (16)
C12—C11—P1—C21	−78.7 (4)	C71—P4—Cu2—S1	−173.35 (14)
C16—C11—P1—C5	−153.9 (3)	C81—P4—Cu2—S1	68.94 (16)
C12—C11—P1—C5	28.5 (4)	C4—P4—Cu2—S1	−54.85 (16)
C16—C11—P1—Cu1	−27.9 (3)	C71—P4—Cu2—I2	66.06 (15)
C12—C11—P1—Cu1	154.6 (3)	C81—P4—Cu2—I2	−51.64 (16)
C26—C21—P1—C11	−160.4 (4)	C4—P4—Cu2—I2	−175.44 (15)
C22—C21—P1—C11	20.6 (4)	C31—P2—Cu2—P4	−176.36 (15)
C26—C21—P1—C5	94.1 (4)	C41—P2—Cu2—P4	59.99 (15)
C22—C21—P1—C5	−84.8 (4)	C5—P2—Cu2—P4	−54.57 (15)
C26—C21—P1—Cu1	−35.9 (4)	C31—P2—Cu2—S1	−49.76 (15)
C22—C21—P1—Cu1	145.2 (3)	C41—P2—Cu2—S1	−173.40 (14)
P2—C5—P1—C11	165.5 (2)	C5—P2—Cu2—S1	72.04 (15)
P2—C5—P1—C21	−88.2 (2)	C31—P2—Cu2—I2	61.10 (15)
P2—C5—P1—Cu1	41.6 (2)	C41—P2—Cu2—I2	−62.55 (15)
C36—C31—P2—C41	122.1 (3)	C5—P2—Cu2—I2	−177.11 (14)
C32—C31—P2—C41	−58.1 (4)	C1—S1—Cu2—P4	−70.66 (16)
C36—C31—P2—C5	−134.9 (3)	Cu1—S1—Cu2—P4	51.30 (5)
C32—C31—P2—C5	45.0 (4)	C1—S1—Cu2—P2	160.97 (16)
C36—C31—P2—Cu2	−7.2 (4)	Cu1—S1—Cu2—P2	−77.07 (4)
C32—C31—P2—Cu2	172.6 (3)	C1—S1—Cu2—I2	49.77 (16)
C42—C41—P2—C31	−5.5 (4)	Cu1—S1—Cu2—I2	171.74 (3)
C46—C41—P2—C31	174.7 (3)		

### Hydrogen-bond geometry (Å, º)

Cg1 is the centroid of the C41–C46 ring.

**Table:**

*D*—H···*A*	*D*—H	H···*A*	*D*···*A*	*D*—H···*A*
N1—H1*A*···I2^i^	0.87 (2)	2.85 (3)	3.645 (4)	152 (4)
N1—H1*B*···I2	0.84 (2)	2.96 (2)	3.796 (4)	171 (5)
N2—H2···I1	0.88 (2)	2.70 (2)	3.563 (4)	168 (4)
C63—H63···S1^ii^	0.95	2.95	3.751 (5)	143
C7—H7*A*···I1^iii^	0.98	3.02	3.961 (7)	161
C14—H14···*Cg*1^iv^	0.95	3.37 (1)	4.08	130

Symmetry codes: (i) −*x*, −*y*, −*z*+1; (ii) *x*, −*y*+1/2, *z*+1/2; (iii) *x*+1, *y*, *z*; (iv) −*x*+1, *y*+1/2, −*z*+3/2.
